# A 3 °C global RCP8.5 emission trajectory cancels benefits of European emission reductions on air quality

**DOI:** 10.1038/s41467-017-00075-9

**Published:** 2017-07-25

**Authors:** A. Fortems-Cheiney, G. Foret, G. Siour, R. Vautard, S. Szopa, G. Dufour, A. Colette, G. Lacressonniere, M. Beekmann

**Affiliations:** 1 Laboratoire Interuniversitaire des Systèmes Atmosphériques, CNRS/INSU UMR7583, Université Paris-Est Créteil et Université Paris Diderot, Institut Pierre Simon Laplace, Créteil, 94000 France; 2 Laboratoire des Sciences du Climat et de l’Environnement, Institut Pierre Simon Laplace, CEA/CNRS/UVSQ, Gif sur Yvette, 91190 France; 3 Institut National de l’Environnement et des RISques industriels (INERIS), Verneuil en Halatte, 60550 France

## Abstract

Despite the international agreement to reduce global warming to below 2 °C, the Intended Nationally Determined Contributions submitted for the COP21 would lead to a global temperature rise of about 3 °C. The relative consequences of such a one-degree additional warming have not yet been investigated for regional air quality. Here we found that a + 3 °C global pollutant emission trajectory with respect to pre-industrial climate (reached along the 2040–2069 period under a RCP8.5 scenario) would significantly increase European ozone levels relative to a 2 °C one (reached along the 2028–2057 period under a RCP4.5 scenario). This increase is particularly high over industrial regions, large urban areas, and over Southern Europe and would annihilate the benefits of emission reduction policies. The regional ozone increase mainly stems from the advection of ozone at Europe’s boundaries, themselves due to high global methane concentrations associated with the RCP8.5 emission scenario. These results make regional emission regulation, combined with emissions-reduction policies for global methane, of crucial importance.

## Abstract (web summary)

Current national pledges to reduce greenhouse gas emissions track to a temperature rise of about 3 °C. Here the authors use future projections to show that 3 °C warming under a business as usual scenario would result in large increases in ozone concentrations, off-setting any benefits from mitigation policies.

## Introduction

Despite a stabilization over the last decade, tropospheric ozone (O_3_) remains a serious environmental problem in Europe^1^, as its concentrations are still far from achieving levels of air quality standards of the European Commission (especially the number of exceedances of the daily maximum 8-h average of 120 μg/m^3^) and of the World Health Organization^2^ set for the protection of human health (maximum daily 8-h average of 100 μg/m^3^). Therefore, further efforts are necessary to improve air quality with respect to ozone: assuming the implementation of current air quality legislation, the European anthropogenic emissions of ozone precursors are expected to decline until 2050^3^. However, climate change can perturb chemical processing, local meteorology, and the long-range transport that influence air pollution^4–12^, and could annihilates benefits of European emission reductions on air quality.

As voluntary contributions of parties to reduce greenhouse gas emissions (INDC, Intended Nationally Determined Contributions) submitted before The Paris 2015 climate conference (COP21) would probably result in a global temperature rise near 3 °C with respect to pre-industrial levels^13^, we investigated here the impact of a + 3 °C climate change (using the RCP8.5 scenario) on future European ozone surface concentrations and compared it to that of a 2 °C warming (using the RCP4.5 scenario). To this end, several future air quality scenarios for Europe were performed with a regional chemistry-transport model (CHIMERE^14^), combined with different global background composition and regional air pollutant emission scenarios in the horizon 2050.

## Results

### Impact of a 3 °C global RCP8.5 emission trajectory

**Fig. 1:**
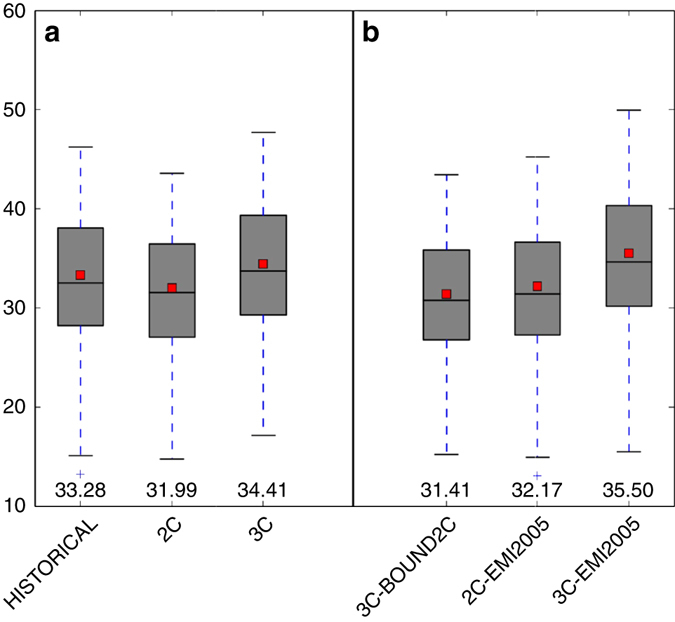
Distribution of annual mean European ozone concentrations. Distribution of annual mean European ozone land concentrations for each of **a** the main scenarios HISTORICAL, 2C, and 3C and **b** the sensitivity tests 3C-BOUND2C, 2C-EMI2005, and 3C-EMI2005, in ppbv. *Filled boxes* indicate the interquartile range, the *whiskers* indicate the full range, the *line* and *square*, respectively indicate the median and the mean. Numbers at the *bottom* of the figure also indicate the means, calculated from the 30-year period of the different scenarios. The different scenarios are described in Table 1

Despite a reduction in anthropogenic emissions in the ECLIPSE-v4a current legislation emissions (CLE) 2050 emissions (decrease by about −67%, −41%, and −49% respectively, for NO_x_, NMVOC, and CO in ECLIPSE-v4a CLE 2050 compared to the 2005 levels), the mean annual surface ozone concentrations for a 3 °C warming (simulation 3C, see Table 1) are about 3.5% higher than the HISTORICAL simulation (annual mean ozone concentrations of 34.41 and 33.28 ppbv, respectively, see Fig. 1). Moreover, with equal regional ozone precursor ECLIPSE-v4a CLE 2050 emissions, mean annual ozone in the 3C simulation is 8% higher than in the 2C simulation. Differences between these scenarios 2C and 3C are statistically significant (means of 31.99 ± 1.01 and 34.41 ± 1.07, respectively).

**Table 1:** Description of the simulations performed by CHIMERE for the different scenarios

Name	Objectives of the simulation	Climate	Boundary conditions^a^	Emissions^b^
HISTORICAL^c^	Baseline scenario	1971–2000	RCP2.6 2005 (330)	2005
2C^c^	Likely O_3_ concentrations in a + 2 °C world	+2 °C	RCP4.5 2050 (305)	2050 CLE
2C-EMI-2005	Sensitivity to emissions	+2 °C	RCP4.5 2050 (305)	2005
3C	Likely O_3_ concentrations in a + 3 °C world	+3 °C	RCP8.5 2050 (350)	2050 CLE
3C-EMI-2005	Sensitivity to emissions	+3 °C	RCP8.5 2050 (350)	2005
3C-BOUND2C	Sensitivity to regional climate	+3 °C	RCP4.5 2050 (305)	2050 CLE

^a^Numbers in brackets refer to the tropospheric ozone burden in Tg associated with the different RCP scenarios^25^

^b^CLE is for current legislation emissions

^c^the HISTORICAL simulation as well as the 2C simulation are described in recent studies^24, 26^

**Fig. 2:**
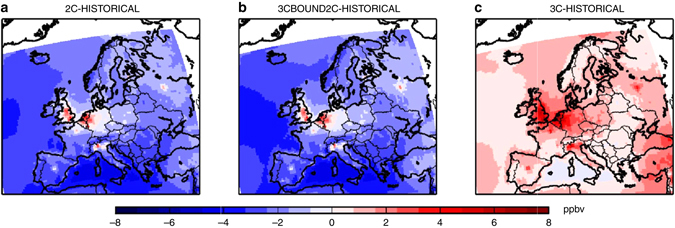
Differences of mean annual ozone concentrations. Differences of mean annual ozone concentrations of **a** 2C minus HISTORICAL, **b** 3C-BOUND2C minus HISTORICAL, and **c** 3C minus HISTORICAL. The means are calculated from the 30-year period of the different scenarios. Units are ppbv. The different scenarios are described in Table 1. This figure has been generated using the Matplotlib library for the Python programming language (https://matplotlib.org/)

Over land, and particularly over industrial regions, over megacities and over Turkey (see the increase of about 4 ppbv in Fig. 2c), mean annual ozone concentrations for a + 3 °C climate would be indeed higher than for both a historical or + 2 °C climate. This means that the efforts made to decrease regional European ozone precursor emissions could be annihilated in the 3C simulation, under the RCP8.5 scenario. As seen in Fig. 1, it should also be noted that, in addition to annual ozone mean, the extremes (both the minimum and the maximum) of the distribution are also increased in the 3C simulation.

**Fig. 3:**
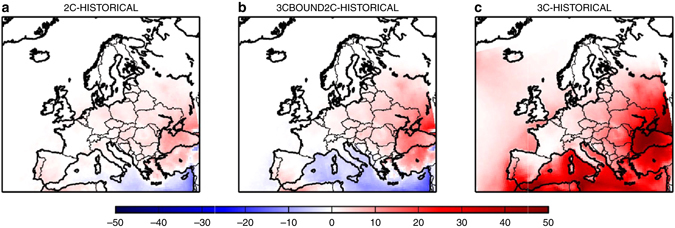
Differences of annual mean number of days exceeding the WHO threshold. Differences of annual mean number of days where summer daily maximum 8-h average ozone concentrations exceeds 50 ppbv, which is a target value for the World Health Organization, for different scenarios: **a** 2C minus HISTORICAL, **b** 3C-BOUND2C minus HISTORICAL, and **c** 3C minus HISTORICAL. The means are calculated from the 30-year period of the different scenarios. Units are ppbv. The different scenarios are described in Table 1. This figure has been generated using the Matplotlib library for the Python programming language (https://matplotlib.org/)

**Fig. 4:**
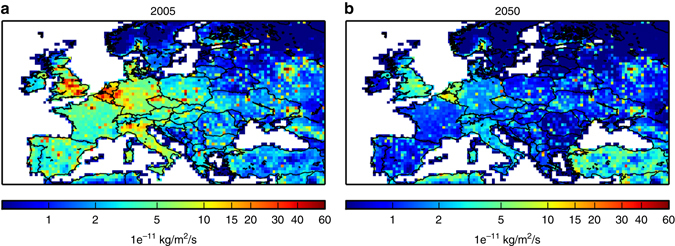
Evolution of ECLIPSE-V4a NO_x_ emissions between 2005 and 2050. ECLIPSE-v4a NO_x_ emissions in **a** 2005 and in **b** 2050. Units are 1e^−11^ kg/m^2^/s. This figure has been generated using the Matplotlib library for the Python programming language (https://matplotlib.org/)

**Fig. 5:**
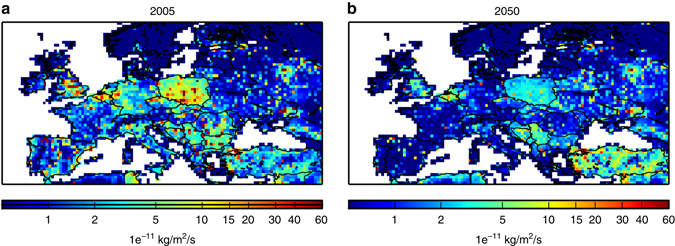
Evolution of ECLIPSE-V4a SO_x_ emissions between 2005 and 2050. ECLIPSE-v4a SO_x_ emissions in **a** 2005 and **b** in 2050. Units are 1e^−11^ kg/m^2^/s. This figure has been generated using the Matplotlib library for the Python programming language (https://matplotlib.org/)

The increase of the ozone maximum can also be seen with the maximum daily 8-h average ozone concentrations (MDA8) during the summer (June–September) period. The annual mean number of days per year when summer MDA8 exceeds 100 μg/m^3^ (equivalent to 50 ppbv), which is a target air quality guideline value for the World Health Organization, is higher both for 2C and for 3C than for the HISTORICAL simulation in South-eastern Europe (see Fig. 3a, c). It should be noted that ECLIPSE-v4a CLE 2050 emissions are predicted to increase over Turkey with respect to the 2005 levels (see Figs. 4 and 5, for NO_x_ and SO_2_ emissions, respectively): the projections show high growth rates in activity and since Turkey lacks stringent laws for stationary combustion, the power plant sector would be responsible for a strong growth in NO_x_ emissions (Klimont. Z., personal communication). The number of ozone exceedance days is much higher for 3C than for 2C in South-eastern Europe and over the Mediterranean sea.

### Understanding the increase of ozone between 2 and 3 °C

**Fig. 6:**
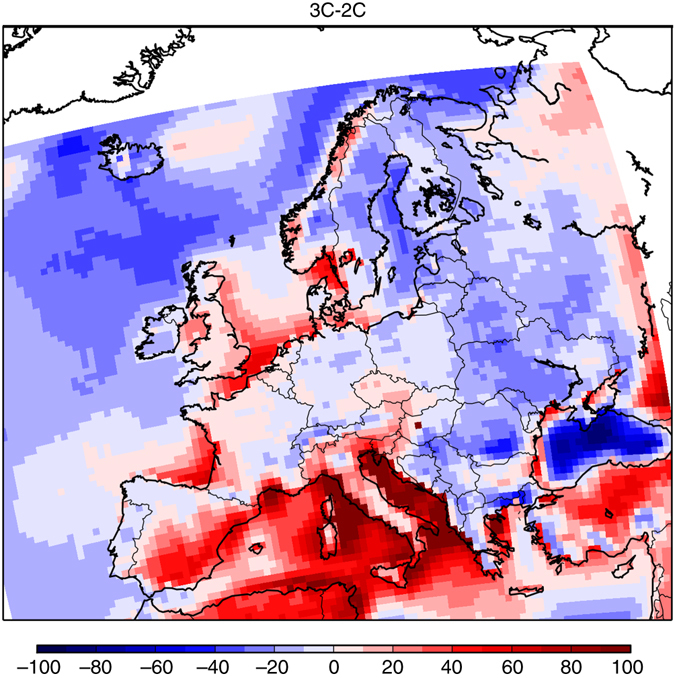
Summer differences of 3C minus 2C for the mean number of hot days when daily maximum temperature is higher than 30 °C. The mean is calculated from the 30-year period of the different scenarios. This figure has been generated using the Matplotlib library for the Python programming language (https://matplotlib.org/)

**Fig. 7:**
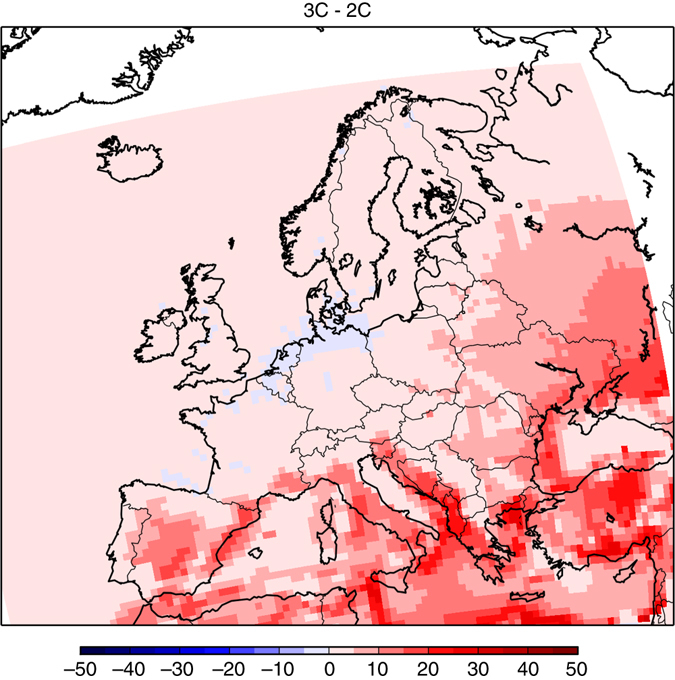
Summer differences of 3C minus 2C for the mean boundary layer height. The mean is calculated from the 30-year period of the different scenarios. Units are meters. This figure has been generated using the Matplotlib library for the Python programming language (https://matplotlib.org/)

This increase of ozone concentrations between 2C and 3C is mostly linked to the boundary conditions under the RCP8.5 scenario. This can be concluded from a simulation 3C-BOUND2C where the 3C simulation is repeated but with boundary concentrations as for the 2C simulation. This simulation shows strong differences in MDA8 exceedance days with the 3C simulation (respectively, Fig. 3c, b), making evident the large impact of boundary conditions (CLE emissions and regional climate input is equal for both simulations). The determining elements of RCP8.5 for the 3C simulation are indeed a doubled global methane concentrations compared to RCP4.5, playing a significant role for ozone concentrations^15^, and a 40–150% greater stratospheric influx of ozone^16^, both leading to an important increase of tropospheric ozone levels. Nevertheless, the regional climate change can also explain a part of this increase in ozone exceedance days in Eastern Europe, with a higher number of hot summer days (i.e., with daily maximum temperature higher than 30 °C, see Fig. 6) and a lower thickness of the boundary layer height (see Fig. 7). It is interesting to note that a decrease of ozone exceedance days, only due to regional climate change, is observed over the Mediterranean sea with the 3C-BOUND2C simulation. This could be related, among others, to a larger thickness of the boundary layer over this region (see Fig. 7) and then to enhanced dilution of ozone and of its precursors.

### Key role of European ozone precursors emissions

Finally, we investigated the role of the assumed decrease of European emissions. For this, we performed simulations using the 2005 emissions and we compared the impact of simulations 2C vs. 2C-EMI-2005, and 3C vs. 3C-EMI-2005, respectively, on ozone concentrations. It should be recalled that 2C and 3C simulations have been performed with ECLIPSE CLE emissions, assuming a significant emission reduction of precursors until year 2050 throughout all of Europe (except over Turkey).

**Fig. 8:**
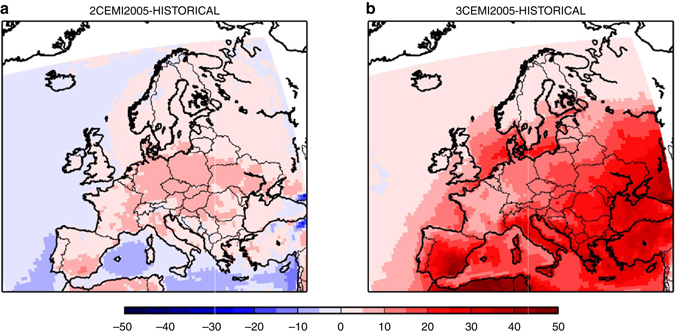
Differences of annual mean number of days where summer daily maximum 8-h average ozone concentrations exceeds 50 ppbv. This is a target value for the World Health Organization, for different scenarios: **a** 2C-EMI2005 minus HISTORICAL, and **b** 3C-EMI2005 minus HISTORICAL. The means are calculated from the 30-year period of the different scenarios. Units are ppbv. The different scenarios are described in Table 1. This figure has been generated using the Matplotlib library for the Python programming language (https://matplotlib.org/)

By keeping present day’s emissions in a future + 2 or + 3 °C climate on a 2005 level, mean summer ozone concentrations would be respectively + 10% (+3.30 ppbv) and + 12% (+4.30 ppbv) larger as for year 2050 ECLIPSE CLE emissions. It should be noted that this increase would not apply to high NO_x_ urban areas where low ozone levels associated to NO titration would persist. Moreover, the number of exceedance days over the WHO threshold (MDA8 > 50 ppb) would be significantly increased if regional ozone precursor emissions would not be reduced. It would be at least 25 days per year at each location in Europe (except over northern countries such as Norway and Finland, see Fig. 8) and could reach about 100 days over the Mediterranean sea in the 3C-EMI2005 simulation. These results demonstrate that the regional emissions decrease projected by the CLE scenario is crucial in order to mitigate the impact of a + 3 °C global trajectory under the RCP8.5 scenario. Nevertheless, such a decrease would not be fully sufficient to prevent an increase of European ozone concentrations.

## Discussion

The benefits of European anthropogenic emission reductions would probably be annihilated over large regions of Europe with a global + 3 °C temperature increase, with ozone background and maximum levels enhanced over Europe compared to the present day. This is due to global changes of climate and background atmospheric composition (with high methane concentrations associated with the RCP8.5 scenario). These results confirm that if European air quality is to be improved, global methane emissions should be regulated providing both positive effects on regional air quality but also on climate change^7, 17^. Also, the predicted CLE regional decrease for ozone precursors in the horizon 2050 remains crucial for European air quality, and particularly for the Southern European population. Over the Mediterranean Sea and adjacent areas, if current emissions were not reduced in a + 3 °C world under the RCP8.5 scenario, the number of ozone exceedance days could reach 100 per year. Considering the adverse effects of short-term exposure to daily ozone concentrations^18, 19^, this would strongly affect both human health and vegetation.

## Methods

### The regional chemistry-transport model CHIMERE

The regional chemistry-transport model CHIMERE^14^ is driven by weather conditions provided by the IPSL-CM5A-MR global climate model simulations^20^ downscaled by the WRF regional climate model as produced for the EURO-CORDEX ensemble^21, 22^. The regional simulation domain used here is a grid of 50 × 50 km encompassing Europe and part of the North-Eastern Atlantic Ocean^23^ and North Africa. Using this model suite and focusing over Europe, we compare future air quality simulations covering 2 °C and 3 °C warming periods (see Table 1) with air quality in a reference climate period (1971–2000). They are 30-year periods encompassing the year for which the global average warming reaches the  + 2 and + 3 °C target, respectively, in the RCP4.5 and RCP8.5 scenarios, a methodology employed in recent studies^21, 24^. For IPSL-CM5A-MR simulations, the 2 °C warming is reached along (2028–2057) under RCP4.5 assumptions and the 3 °C warming along (2040–2069) under RCP8.5 assumptions. Both periods correspond to the middle of the century, for which dedicated regional air pollutant scenarios have been constructed (ECLIPSE-v4a^22^, http://eclipse.nilu.no). We used here regional air pollutant emissions corresponding to application of current legislation, projected for 2050 (CLE 2050 emissions). Average climatological boundary concentrations of ozone and precursors were taken from Laboratoire de Météorologie Dynamique- INteraction with Chemistry and Aerosols (LMDz-INCA) global simulations^25^ for the specific periods and scenarios.

### Climatological boundary conditions from LMDz-INCA

The lateral and upper boundary conditions were indeed computed with the global climate-chemistry LMDz-INCA model^25^. The monthly-mean climatologies correspond to 11-year means since the interannual variability in the LMDz-INCA simulation, driven by climate variability, has no reason to match in time the variability of the climate projection used in the present work. The climatologies are centered on 2006 for the present and 2050 for the future assuming the RCP4.5 and RCP8.5 emissions and climate scenario at the global scale. These future scenarios induce, respectively, a −1 ppbv decrease and a + 21 ppb increase of the mean global surface ozone at the 2050 horizon compared to 2005. This ozone response of LMDz-INCA is close to the multi-model ensemble mean in the ACCMIP intercomparison projet^16^.

### Set-up of the scenarios

All configurations corresponding to the seven experiments used in this study are described in Table 1. Simulations of historical climate (between 1971 and 2000), i.e., the HISTORICAL simulation as well as the 2C simulation (between 2028 and 2057) are described in recent studies^24^ ^, 26^. The HISTORICAL simulation, for which monthly ozone fields were reasonably close to Airbase measurements^26^, serves as a baseline for comparison with future ozone concentrations (under + 2 and + 3 °C climate and different emissions scenarios). The 2C simulation was performed with projected ECLIPSE CLE emissions^3^ (http://eclipse.nilu.no) and with RCP4.5 forcing and associated boundary conditions using LMDz-INCA. The CLE emissions, assuming the implementation of current air quality legislation, show reductions in all anthropogenic air pollutants (except for ammonia). Among scenarios performed in our study, the 2C-EMI-2005 simulation is the same as 2C but with 2005 emission giving information on the role of European emissions mitigation in a + 2 °C climate. 3C is the key simulation of our study showing projected ozone levels in a + 3 °C climate (and corresponding boundary conditions) taking into account current European policy (with 2050 CLE emissions). It should be noted that in this RCP8.5 scenario the methane burden is more than doubled from 2000 to 2100, whereas 2100 burdens are 30%, 10%, and 2.5% lower than 2000 for the RCP2.6, RCP4.5, and RCP6.0, respectively^16^. The RCP8.5 scenario can be probably seen as representative of an upper limit scenario for methane concentrations. Nevertheless, it should be noted that the evolution of atmospheric methane over the last three years closely follows the RCP8.5 scenario^27^. We also have run the 3C-EMI-2005 simulation similar to 2C-EMI-2005 but for the + 3 °C climate. Finally, the 3C-BOUND2C simulation was performed to determine the impact of global scale change (between + 2 and + 3 °C) on European ozone surface concentrations. It is similar to 3C but uses chemical boundary conditions from LMDz-INCA simulations corresponding to the + 2 °C period and the RCP4.5 scenario.

### Consistency between CLE emissions and RCP scenarios

Over our European domain, NO_x_, NMVOC, and CO ECLIPSE-v4a emissions decrease by about −67%, −41%, and −49%, respectively, in 2050 compared to the 2005 levels. It should be noted that these strong reductions are broadly consistent with both the RCP4.5 and the RCP8.5 emission scenarios (decrease by −63%, −50%, −82% for RCP4.5 and −55%, −58%, −70% for RCP8.5, respectively). The evolution of ECLIPSE-v4a methane emissions is in agreement with the RCP4.5 emission scenario (decrease of about 16% in 2050 compared to 2005), but not with the RCP8.5 scenario, which predicts an increase of about 18% in 2050 compared to 2005 levels. However, because of its low reactivity and long lifetime, methane emitted over Europe mostly contributes to O_3_ formation mainly outside of the domain and this inconsistency between RCP8.5 and CLE emission scenarios will not have any impact on the robustness of this study.

### Code availability

The CHIMERE code is available here: www.lmd.polytechnique.fr/chimere/

### Data availability

The ECLIPSE-v4a emissions are available here: http://eclipse.nilu.no
